# Critical congenital heart disease: contemporary prenatal screening performance and outcomes in a multi-centre perinatology service

**DOI:** 10.1186/s12884-024-06350-0

**Published:** 2024-02-24

**Authors:** Fiona Cody, Orla Franklin, Nicola Mc Cay, Zara Molphy, Patrick Dicker, Fionnuala M. Breathnach

**Affiliations:** 1 Department of Obstetrics and Gynaecology, Royal College of Surgeons in Ireland (RCSI, Dublin, Ireland; 2 Department of Obstetrics and Gynaecology, Rotunda Hospital, Parnell Square East, Dublin, D01P5W9 Ireland; 3 Department of Paediatric Cardiology, Children’s Health Ireland CHI@Crumlin, Dublin, Ireland; 4 Department of Paediatrics, Trinity College Dublin, Dublin, Ireland; 5 School of Population Health, Royal College of Surgeons in Ireland (RCSI), Dublin, Ireland

**Keywords:** Prenatal diagnosis, Fetal echocardiography, Ultrasound, Critical congenital heart disease, Obstetrics

## Abstract

**Background:**

Prenatal detection of critical congenital heart disease (CCHD) optimises perinatal decision-making and neonatal outcomes. The objective of this study was to determine the prenatal screening performance, care pathways and perinatal outcomes for prenatally and postnatally diagnosed cases of CCHD over a four-year period.

**Study design:**

This retrospective cohort study in a tertiary centre and its two affiliated secondary sites examined all cases of CCHD, including cases of pregnancy termination and in-utero fetal death, neonatal death and liveborn babies that underwent cardiac catheterization or surgery in the first six weeks of life. Prenatal and postnatal data were ascertained from the first trimester assessment for all patients diagnosed prenatally. Cases requiring intervention that were first identified in the postnatal period were included to determine prenatal detection rates. Follow-up for all cases of CCHD continued to one year of age.

**Results:**

In a consecutive cohort of 49,950 pregnancies in a 4-year period 01/2019 to 12/2022, a prenatal diagnosis of CCHD was made in 96 cases, yielding a prevalence of 1.9 per 1000 births. The prenatal detection for right duct-dependant heart pathology and congenital heart block was 100%, 85% for left duct-dependant pathology and 93% for transposition of the great arteries (TGA). In the prenatally diagnosed group, 37% of cases were complicated by extracardiac structural abnormalities, a genetic diagnosis or both. All cases of prenatal detection were identified in the context of routine anatomy screening rather than specialist Fetal Cardiac screening services. Almost half of all pregnancies complicated by CCHD did not undergo neonatal cardiac intervention, by virtue of parental choice determined either prenatally or after birth. An additional eight babies were diagnosed with CCHD in the neonatal period, such that the prenatal detection rate for CCHD was 92% (96/104, 95% CI = 84%-96%). Survival at 1-year for infants deemed suitable for CCHD surgery was 85%.

**Conclusion:**

In a large unselected population, optimal rates of prenatal detection of critical congenital heart disease can be achieved by a protocolised approach to mid-trimester fetal anatomy ultrasound, underpinned by a programme of sonographer education and training. The cardiac abnormalities most likely to evade prenatal detection are left-sided obstructive lesions.

## Background

Congenital heart disease (CHD) has a prevalence of 4–13 per 1000 live births and is the most common structural congenital anomaly [1]. Approximately 30% of cases of congenital heart disease are considered critical, requiring urgent intervention (surgical or catheter-based) in the early neonatal period [2, 3]. Survival and neurodevelopmental outcome are dependent on timely prenatal diagnosis and input from fetal, neonatal and cardiology services. Global prenatal detection rates remain variable, with reported screening performance ranging from 13% detection in the Slovak Republic to 87% in Northern France [4] and are influenced by availability of ultrasound expertise and access to fetal medicine and fetal cardiology services [5–7]. Where national anatomy screening policies are introduced and/or targeted fetal cardiac training provided, studies have demonstrated substantial improvements in prenatal detection, up to 59% nationally and up to 91% in targeted training studies [2, 8, 9]. This study sought to determine contemporary prenatal detection rates and survival for critical congenital heart disease (CCHD) managed in a designated hospital group that includes a large tertiary perinatology centre and two affiliated regional secondary sites.

## Methods

This retrospective cohort study examined all cases of duct-dependent CCHD requiring or expected to require cardiac intervention in the first six weeks of life among pregnancies managed in a single hospital group. This study was approved by ethics boards across all institutions involved. The Rotunda Hospital, Dublin is a tertiary care centre that offers a fetal cardiology service in collaboration with the single National Pediatric Cardiac Centre that provides care to all cases of CCHD delivered in Ireland. Prenatal ultrasound is performed by qualified sonographers who participate in ongoing training in fetal echocardiography. This study examined the care pathways from early diagnosis to neonatal life over a 4-year period among a cohort of infants with critical congenital heart disease.

All cases of CCHD that underwent pregnancy termination, died in-utero or in the early neonatal period were also ascertained. The protocol in this tertiary care prenatal screening service and its affiliated sites included a dating ultrasound scan at 12–14 weeks’ gestational age, a fetal anatomy screen at 18–22 weeks and a repeat screening ultrasound for cases of non-acquisition of views sufficient to constitute a normal fetal cardiac examination. The following pre-specified cardiac screening views were required for prenatal clearance of the fetal heart: 4-chamber heart view, left and right outflow tract views, three vessel and three vessel tracheal views. Colour assessment was not mandated for the screening examination. In addition to the standard screening cardiac examination at 18–22 weeks, patients attending any of the study centres who had identified risk factors for CHD were offered targeted fetal echocardiography according to criteria that align with prior ISUOG [10] and updated ISUOG [11] and AIUM [12] practice guidelines. Targeted examination of the fetal heart included the use of colour flow Doppler to evaluate inlet and outlet valves, determination of systemic and pulmonary venous return and extended sagittal cardiac views.

Referral was made to the tertiary Fetal Medicine service in the event of non-acquisition of cardiac views on a second attempt by the screening sonographer. All cases of suspected CCHD were referred to the Fetal Medicine service, offered genetic testing where appropriate and underwent detailed fetal echocardiography with a dedicated fetal cardiac team, with Fetal Medicine and Paediatric Cardiology expertise. In the event of multiple congenital anomalies, confirmed genetic abnormality or isolated CCHD associated with significant long-term morbidity, pregnancy termination was discussed. In the setting of an anticipated lethal prognosis, pregnancy termination was offered in accordance with Irish Law. For all cases of prenatally recognised CCHD, a multidisciplinary perinatal plan was determined.

All cases of CCHD delivered in Ireland undergo treatment in a single national centre. Data sources included dedicated prenatal ultrasound and pediatric cardiology databases, perinatal, neonatal, and pediatric chart review, and examination of perinatal mortality reports from the tertiary care centre. Individual cases were traced back to delivery records and birth registration where necessary.

Data management and data quality checks were performed using SAS V9.2. Basic summary statistics were described for the study population. An exact 95% confidence interval was calculated for the overall prenatal detection rate.

## Results

**Fig. 1:**
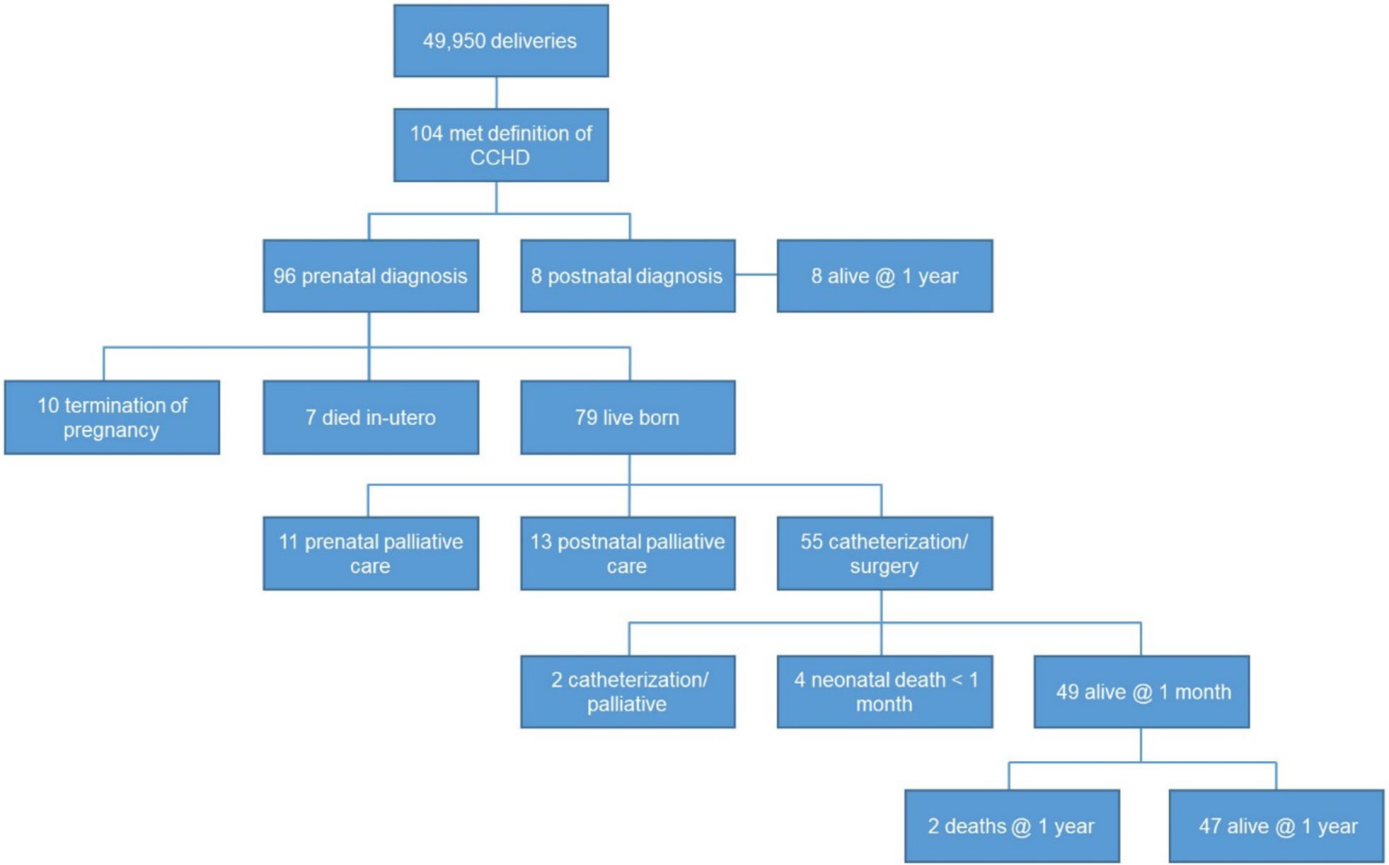
Pregnancy outcomes for CCHD in 3 maternity centres 2019–2022 * Following neonatal cardiac catheterisation, two cases of prenatally-identified CCHD were deemed unsuitable for surgery. A palliative care pathway was instituted thereafter

From January 2019 to December 2022, among a consecutive cohort of 49,950 pregnancies, 202 (0.4%) cases of CHD were identified, of whom 104 were considered to meet the definition for critical congenital heart disease (CCHD), expected to require intervention within the first six weeks of life, for a population incidence of 2 per 1000 pregnancies (Fig. 1).

**Fig. 2:**
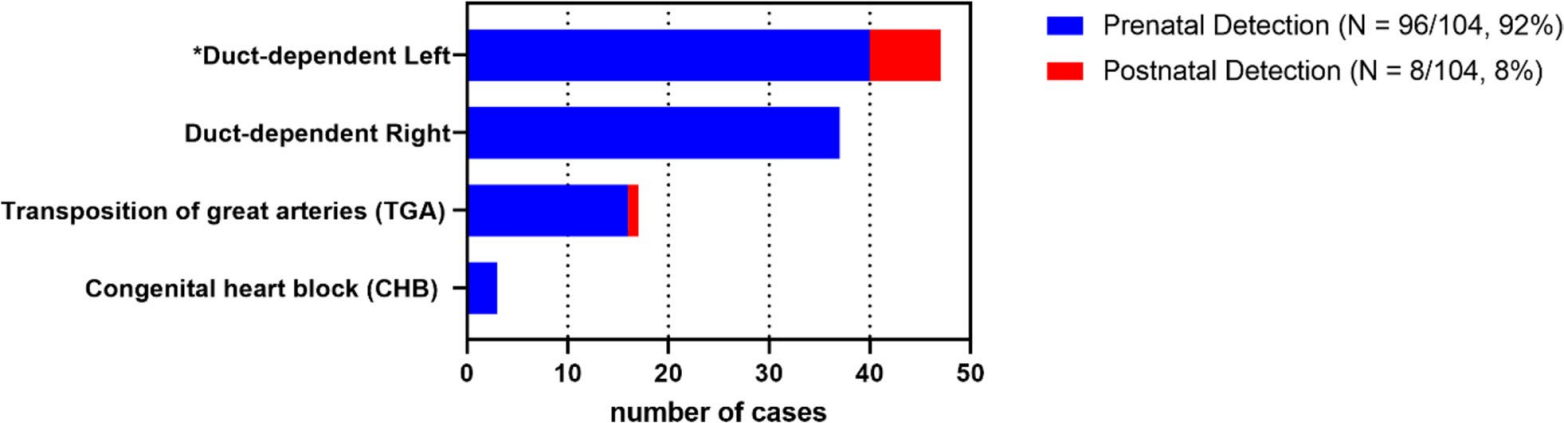
Critical Congenital Heart Disease identified in screened cohort* *N* = 104 *Duct-dependent left (postnatal cases: N = *7* *)* *. 1* = *HLHD. 2* = *Coarctation of the aorta. 1* = *Coarctation of the aorta with Atrioventricular septal defect. 1* = *Coarctation of the Aorta with Shone’s complex. 1* = *Critical aortic stenosis. 1* = *Double Inlet left ventricle (DILV)/TGA/VSD*

Within the cohort of 104 CCHD cases, 96 were diagnosed in the prenatal period, for a prenatal detection rate of 92% (95% CI: 84%—96%). The 96 prenatal cases represented a prevalence of 1.9 per 1000 births over the study. The prenatal cases are presented in Fig. 2 and are categorized into four groups:

1. Left duct-dependant heart disease (Hypoplastic left heart disease, critical mitral valve stenosis, critical aortic stenosis/atresia, Shone complex, interrupted aortic arch, aortic coarctation)
2. Right duct-dependant lesions (Hypoplastic right heart, tricuspid stenosis, critical pulmonary stenosis/atresia, Tetralogy of Fallot with critical pulmonary stenosis)
3. Transposition of the great arteries (TGA)
4. Complete congenital heart block (CHB)

No case of isolated total anomalous pulmonary venous drainage (TAPVD) was observed during the study period, an observation consistent with the rarity of isolated TAPVD diagnosis in the literature [13]. There were three cases of TAPVD identified in association with other complex critical cardiac disease, namely right duct-dependent pathology. In the event of multiple structural cardiac anomalies, cases were classified according to the most critical or duct-dependant anomaly. Comparative prenatal and postnatal detection rates for this CCHD classification are presented in Fig. 2.

Maternal demographic data for all cases of CCHD are presented in Table 1, which demonstrates a mean gestational age at prenatal detection of 22.2 weeks (range 15 to 34 + 4 weeks). Eight percent of cases were significant for a family history of congenital heart disease with one percent of patients having a pre-pregnancy diagnosis of diabetes. All CCHD cases with pre-existing risk factors for CCHD were identified by the routine anatomy screening programme. 33% of CCHD cases (34/104) received prenatal screening in a regional centre, of whom 15% (5/34) were not detected until the postnatal period. This screening performance compares with 4% (3/70) of tertiary-care cases evading prenatal diagnosis.

**Table 1:** Characteristics: Prenatal diagnosis of CCHD (*N* = 96)

Demographic	All (***N***=96)	Lt duct dependant heart (***N*** =40)	Rt duct dependant heart (***N***=37)	Transposition of the great arteries (***N***=16)	Congenital Heart Block^**a**^/ (***N***=3)
Maternal age (years)	32.6 (6.5)	32.8 (6.4)	33.8 (6.1)	28.6 (6.5)	37.3 (3.2)
Maternal BMI^b^ (kg/m^2^)	26.6 (5.6)	27.8 (6.5)	25.3 (4.4)	26.1 (5.3)	30.4 (6.1)
Maternal ethnicity					
White Irish	58 (60%)	25 (63%)	22 (59%)	10 (63%)	1 (33%)
White European	19 (20%)	9 (23%)	7 (19%)	3 (19%)	0
Black African	6 (6%)	3 (8%)	1 (3%)	2 (13%)	0
Asian	8 (8%)	1 (3%)	5 (14%)	0	2 (67%)
Mixed race/other	5 (5%)	2 (5%)	2 (5%)	1 (6%)	0
Gestational age at prenatal diagnosis	22.4 (3.2)	23.0 (4.1)	21.7 (2.0)	22.2 (1.6)	25.8 (6.3)
Family History of CHD	9 (9%)	3 (8%)	6 (16%)	0	0
Referral from secondary/ regional centre	28 (29%)	18 (45%)	7 (19%)	2 (13%)	1 (33%)
Diabetes	1 (1%)	1 (3%)	0	0	0

Categorical data presented as n (%), numeric data presented as mean (SD)

^a^CHB: Congenital heart block

^b^BMI: Body Mass Index in kilograms/metres2

63% of CCHD cases in this cohort occurred in isolation, with the remaining cases (37%) complicated by an extracardiac structural or genetic abnormality.

Perinatal outcomes for 96 cases following prenatal diagnosis are presented in Table 2. A decision to terminate the pregnancy was taken by patients in 10 cases, some of whom met criteria for pregnancy termination in Ireland (legally permissible in the setting of a lethal prognosis). Spontaneous in-utero fetal death occurred in 7% (7/96) (Table 2). Among 79 live-born infants, 11 patients had a palliative care plan in place prenatally. Among the remaining infants considered for surgery, 62 infants were transferred to the Cardiac centre on day 1–14 of life, with prostaglandin administered where needed for the purposes of maintaining ductal patency in accordance with the pre-specified fetal cardiology plan (Table 3).

**Table 2:** Prenatal ciagnoses and outcomes (*N* = 96)

Outcome	All (N=96)	Lt duct dependant heart (N =40)	Rt duct dependant heart (N=37)	TGA (N=16)	Congenital Heart Block(N=3)
Extracardiac structural anomaly^a^	31 (32%)	16 (40%)	14 (38%)	1 (6%)	0
Genetic abnormality					
T21	2 (2%)	0	2 (5%)	0	0
T18	3 (3%)	3 (8%)	0	0	0
T13	1 (1%)	1 (3%)	0	0	0
Di George	4 (4%)	1 (3%)	3 (8%)	0	0
Other^b^	8 (8%)	5 (13%)	3 (8%)	0	0
Isolated CCHD	60 (63%)	22 (55%)	20 (54%)	15 (94%)	3 (100%)
Pregnancy termination	10 (10%)	8 (20%)	1 (3%)	1 (6%)	0
In-utero fetal death	7 (7%)	2 (5%)	4 (11%)	0	1 (33%)
Liveborn	79 (82%)	30 (75%)	32 (86%)	15 (94%)	2 (67%)

n (%) is presented for categorical data and mean (SD) for numeric data

^a^Extra-cardiac anomalies = dysplastic kidneys, solitary kidney, polyhydramnios, Hypoplastic nasal bone, short long bones, duodenal atresia, talipes, Heterotaxy syndrome, spina bifida, echogenic bowel, agenesis of cerebellar vermis, cleft lip, TTTS, bipedal odema

^b^Other genetic diagnosis +  +  = Alagille syndrome,47XXX, Jacobsen syndrome, del ch11q 24.3, monosomy X, copy loss Ch9, Gain Ch11, cri du chat, copy loss Ch16, deletion on Ch.X xq26.1q26.2, tetrasomy of Ch18, T16, tuberous sclerosis, unbalanced karyotype 10, mosaic ring 13 53mb deletion, microdeletion of ch 17

**Table 3:** Neonatal Outcomes (*N* = 79)

Outcome	All (*N* = 79)	Lt Duct Dependant heart (*N* = 30)	Rt Duct Dependant heart (*N* = 32)	TGA (*N* = 15)	Congenital Heart Block (*N* = 2)
Gestational age at delivery	38.3 (1.7)	38.5 (1.9)	38.2 (1.5)	38.4 (1.8)	37.1 (0.4)
Birthweight	3.1 (0.7)	3.1 (0.7)	3.0 (0.6)	3.2 (0.6)	2.3 (0.0)
Palliative care prenatally	11 (14%)	6 (20%)	5 (16%)	0	0
Palliative care postnatally^b^	15 (19%)	6 (20%)	8 (25%)	1 (7%)	0
Prostin given	48 (61%)	16 (53%)	17 (53%)	15 (100%)	0
Transferred to Crumlin	62 (78%)	22 (73%)	23 (72%)	15 (100%)	2 (100%)
Day of transfer	2.1 (4.0)	2.1 (2.9)	2.9 (5.9)	1.3 (0.8)	1.0 (0.0)
Urgent cardiac catheterization	36 (46%)	9 (30%)	19 (59%)	8 (53%)	0
Surgery performed	53 (67%)	17 (57%)	21 (66%)	13 (87%)	2 (100%)
Surgery or catheterisation	55 (70%)	17 (57%)	23 (72%)	13 (87%)	2 (100%)
Surgery and catheterisation	33 (42%)	9 (30%)	16 (50%)	8 (53%)	0
Duration of 1st hospital stay	24.1 (32)	20.6 (28)	23.0 (39)	30.9 (31)	17.0 (4.2)
Survival at 28 days post-catheterisation/surgery^a^	49(89%)	16 (94%)	19 (83%)	12 (92%)	2 (100%)
Survival at 1-year post-surgery^a^	47(85%)	15 (88%)	18 (78%)	12 (92%)	2 (100%)

n (%) is presented for categorical data and mean (SD) for numeric data

^a^Denominator = infants who underwent surgery/catheterisation (*N* = 55)

^b^Some babies were deemed palliative postnatally after evaluation in cardiac care unit or after catheterisation

 A 94% detection rate (16/17) for TGA was observed with a 92% survival rate at one year. The three TGA cases that did not survive comprised of one case of complex TGA (with a genetic diagnosis) where the patient chose pregnancy termination, one case deemed unsuitable for surgery by virtue of severe inlet valvular dysfunction and one perioperative neonatal death. All three cases of congenital heart block were detected prenatally, one fetus died in utero with the remaining two infants alive at 12 months. At one year of age, an 88% survival rate for left-sided duct-dependant lesions and 78% for right-sided duct-dependant cases was observed.

The eight postnatally-diagnosed CCHD cases are described in Fig. 1. There were two cases of single-ventricle pathology undetected by the prenatal screening programme. One had hypoplastic left heart disease (HLHD) and one had double inlet left ventricle (DILV) with TGA and aortic coarctation. Both cases delivered in secondary, regional centres. 62% (5/8) of CCHD cases that were not identified prenatally received prenatal care in regional sites. 87% (7/8) of cases diagnosed postnatally were left-sided duct-dependant lesions (Fig. 1). All cases of single ventricle pathology, TGA and CHB in the tertiary care centre were identified by the screening programme.

## Discussion

Prenatal detection of CCHD makes a compelling case for targeted screening in the prenatal period owing to its complexity and the time-sensitive nature of neonatal intervention. A neonatal mortality risk of up to 50% is reported in the setting of neonatal hospital discharge with undetected CCHD [14]. An Irish study in 2017 reported a one-year mortality rate for CCHD that was tenfold higher in babies diagnosed postnatally compared to CCHD cases born with a prenatal diagnosis [6].

A meta-analysis of eight studies reported a survival advantage and lower levels of neonatal morbidity for CCHD diagnosed in the prenatal period [15]. Although the use of pulse oximetry as a postnatal screening tool can help identify CCHD not detected by prenatal screening, many new-borns with CCHD do not develop clinically appreciable hypoxaemia until after discharge from hospital [16]. Some critical lesions such as hypoplastic left heart disease may present with significant cardiovascular compromise without apparent cyanosis [16]. Therefore, optimal timely detection of CCHD remains heavily reliant on prenatal diagnosis.

An undeniable paradox of prenatal screening is that cases at the greatest disadvantage from non-detection in the prenatal period, namely those where delivery is planned in a centre remote from the pediatric cardiac service, are those for whom access to optimal screening expertise may be less readily available [17–19].

The extent to which specialist fetal echocardiography for selected pregnancies considered to be at heightened risk for congenital heart disease may result in an appreciable increase in prenatal detection is unclear. The vast majority of cases of CCHD in this study had no risk factors for CHD. Furthermore, the study period was notable for a 12-month period (March 2020 to February 2021) during which the COVID-19 pandemic-related rationalisation protocols were introduced to limit patient-facing exposure. During that time, all fetal cardiac screening was limited to the mid-trimester fetal anatomy examination, with no supplementary imaging offered to women with identified risk factors for CHD. The examination time was also reduced in the pandemic from 30 to 15 min for routine ultrasound examination as a means of reducing sonographer exposure to viral risk. These constraints did not have a detrimental effect on prenatal detection of CCHD. Indeed, throughout the four-year study period, no case of CCHD was detected by targeted screening in the selected population. This observation suggests that the prenatal detection of CCHD does not rely on the provision of targeted supplementary examinations for pregnancies perceived to be at heightened risk. Rather, excellent prenatal detection rates can be achieved women perceived to be at with a protocolised screening service and an established sonographer training programme.

This study was conducted in one of the busiest maternity hospitals in Europe, with two additional affiliated referral centres, over a four-year period. The existence of a single National Pediatric Cardiac Centre in Ireland, participating in an externally validated Pediatric Cardiac data collection system that is submitted in real time, results in complete ascertainment of all cases of CCHD evaluated for surgical and catheter intervention within the first six weeks of life. In addition, all cases of fetal abnormality identified in the two regional sites, including all cases of CCHD, are referred to the tertiary Fetal Medicine unit, which maintains a contemporaneous record of all cases of CCHD identified in all three centres. No case of pregnancy termination within Ireland for CCHD proceeds without referral to the centralised fetal cardiac service. All cases of in-utero fetal death in the context of fetal abnormality are recorded and reported in the Annual Report of the tertiary centre [20].

Postnatally diagnosed cases in the event of infant mortality are reported to the cardiac database by the paediatric pathology service centralised in CHI (Children’s Health Ireland)@Crumlin. Therefore, this study should be considered to reflect complete ascertainment of all cases of CCHD identified in the prenatal period and diagnosed in the neonatal period during the four-year study timeframe across all three contributing centres. The prevalence of CCHD in this population was 1.9 per 1000 live births, which is aligned with the prevalence seen in other series. In 2018, Bakker et al. reported a prevalence of 19.1 per 10,000 births in a retrospective cohort study across 15 international centres in Europe, North America and Asia [4].

In 2016, the Irish National Maternity Strategy 2016–2026 devised an implementation plan of action aimed at improving services across all 19 maternity units in Ireland. This included a plan for provision of a routine first trimester ultrasound examination at 10–14 weeks’ gestation and a mid-trimester anatomy scan (18–22 weeks) to all pregnant women [21]. Prior to the strategy, 7/19 units at that time were providing a mid-trimester anatomy scan, 7/19 units were not providing the service and 5/19 units provided the service to select groups of patients [6]. A national detection rate of 57% was reported in 2019, for CCHD in Ireland with 71% reported in centres with an established universal screening protocol [22].

Retrospective population studies have emphasised the importance of national screening programmes and protocols for improvement in the prenatal detection of CCHD [23, 24]. In the Netherlands, the largest reported population screening programme for CCHD to date resulted in an increase in prenatal detection from 36 to 60% in 2007 [23]. In Sweden, where a national screening programme was introduced between 2014 and 2017, the overall detection rate for CCHD was reported as 59%. While the detection of single ventricle pathologies was reported at 100%, only 9% of TGA cases were detected during pregnancy and the prenatal detection of aortic coarctation was 18% [24]. A National anatomy screening programme in the UK has been in place for 25 years and updated Fetal Anatomy Screening Programme (FASP) guidelines were introduced in 2015 with the aim of increasing detection rates to 50%. The reported national prenatal detection rate for CHD in the UK is 30–50% among infants undergoing catheterisation or surgery [25]. Importantly, published data that do not include fetal deaths, pregnancy terminations, pre-intervention neonatal death or palliative care cases will inevitably reflect an under-representation of both CCHD prevalence and the performance of prenatal screening programmes.

Targeted fetal echocardiography training programmes have been shown to yield significant improvements in prenatal detection. Following implementation of a training programme CHD detection rates of 91% (21/23) were reported in a review of 5445 fetal anatomy screening ultrasound examinations over a two-year period in a single unit in the UK [9]. Comparable success was reported by The Rotunda Hospital, Dublin programme, where targeted sonographer training resulted in an increase in the prenatal detection of major CHD from 31% in 2010 to 91% in 2012 [8]. Suard and colleagues [26] recently reported a prenatal detection rate of 71% in southern France across two large centres, with 97% of single ventricle abnormalities and 80% of TGA cases identified prenatally. Evans improved overall CHD detection rates from 36–71% in Nevada through implementation of training and multidisciplinary support from fetal medicine and cardiology teams between 2012 and 2014 [19]. His later paper in 2019 reported a further increase in overall CHD detection rates to 78% [27].

It is worth noting that the tertiary centre performed better at prenatal detection of CCHD during the study period. The achievement of performance standards in regional/ peripheral sites equivalent to those observed in tertiary care university centres is a challenge that prevails in all areas of healthcare [17]. While the regional sites in this study have access to the same standard of equipment and with a comparable sonographer workload, readiness of available on-site MFM expertise in the tertiary centre represents the most glaring service disparity. Enhancement of MFM staffing presence at regional centres, in addition to the sharing of practice protocols and the introduction of educational initiatives in fetal echocardiography are progressing since this study period.

A national US study in 2020 highlighted variable levels of education among sonographers as a contributory factor to poor prenatal detection [5]. In Ireland, all practitioners involved in the provision of fetal cardiac ultrasound services are qualified sonographers with a background in either midwifery or radiography and all hold a postgraduate qualification in obstetric ultrasound. In 2021, 86/157 (55%) of sonographers in Ireland held a Master’s degree qualification in ultrasound [28]. The majority of studies reporting on the performance of prenatal screening programmes do not provide information relating to the competencies, experience or qualifications held by sonographers [29].

The provision of on-site Fetal Medicine support was cited as an independent contributor to improved prenatal detection in a Spanish study [7]. Equally, Pinto’s 2020 national report associated the absence of fetal medicine support in some US centres with low rates of prenatal detection of CCHD [5].

Periodic training updates, revision of screening protocols and ongoing sonographer support from fetal medicine and fetal cardiology services have continued since The Rotunda Hospital Cardiac Screening Programme was established in 2010, with extension of the screening protocol to implementation in two regional centres, including on-site fetal medicine support at both regional centres.

Among almost 50,000 deliveries in this hospital group during the four-year study period, eight cases of unanticipated CCHD that required urgent intervention were managed at the National Pediatric Children’s Heart Centre. A retrospective review of prenatal imaging was beyond the scope of this audit, but is planned for a future study, subject to Institutional Ethics Board approval.

A limitation of the study is that not all perinatal deaths at any participating hospital underwent post-mortem examination, nor did all pregnancy terminations undergo post-mortem confirmation of a prenatal diagnosis. While all cases of perinatal mortality in the tertiary centre were ascertained by way of an institutional perinatal mortality report that describes each perinatal death, and all cases were examined for prenatal or neonatal features consistent with CCHD, it is possible that a perinatal death in one of the regional centres may have featured CCHD and was not ascertained by this review. Post-mortem confirmation of CCHD among cases that underwent termination was not routinely undertaken. However, all cases of CCHD that proceeded to TOP were subjected to review by two MFM specialists, a paediatric cardiologist and with multidisciplinary review of imaging both at MFM and Paediatric Cardiothoracic fora in order to eliminate diagnostic uncertainty. A further limitation is that the low population prevalence of CCHD inevitably results in wide confidence intervals surrounding the detection rate estimates.

The termination of pregnancy (TOP) rate in the setting of CCHD was low (10%) in this cohort and the palliative care rate high (26%). Since 2018, pregnancy termination in Ireland is legally permissible in the setting of a diagnosis that is expected to lead to death within the first 28 days of life [30]. However, some parents opt for perinatal palliation, particularly where societal or cultural attitudes to pregnancy termination prevail in spite of the recent removal of legal constraints. The low termination rate had the unintended effect of maximising the opportunity for postnatal confirmation.

## Conclusion

To our knowledge, the overall 92% prenatal detection rate for CCHD reported in this population, comprising a single large tertiary and two affiliated regional maternity sites, is the highest prenatal detection rate reported in the literature to date in an unselected population of this scale. Our data illustrate that optimal rates of prenatal detection can be achieved by a protocolised approach to mid-trimester fetal anatomy ultrasound in all pregnancies, underpinned by a programme of sonographer education and training. While led by the tertiary centre, this approach should include all teams in referring regional centres. It is notable that all detected cases of CCHD were identified by sonographers performing routine anatomy screening and not in specialist cardiac screening clinics. This is a welcome finding, as CCHD typically occurs in pregnancies that at the outset would not be identified as high risk for CCHD. The review confirms that left heart obstruction lesions remain the Achilles Heel of prenatal cardiac imaging.

## Data Availability

No datasets were generated or analysed during the current study.
